# Exceptional Lithography Sensitivity Boosted by Hexafluoroisopropanols in Photoresists

**DOI:** 10.3390/polym16060825

**Published:** 2024-03-15

**Authors:** Junjun Liu, Dong Wang, Yitan Li, Haihua Wang, Huan Chen, Qianqian Wang, Wenbing Kang

**Affiliations:** National Engineering Research Center for Colloidal Materials, School of Chemistry and Chemical Engineering, Shandong University, Jinan 250100, China; junjun-liu0710@outlook.com (J.L.); dongwang@sdu.edu.cn (D.W.); yitanli@sdu.edu.cn (Y.L.);

**Keywords:** hexafluoroisopropanol, high sensitivity, photoresist, photoacid generation

## Abstract

Advanced lithography requires highly sensitive photoresists to improve the lithographic efficiency, and it is critical, yet challenging, to develop high-sensitivity photoresists and imaging strategies. Here, we report a novel strategy for ultra-high sensitivity using hexafluoroisopropanol (HFIP)-containing fluoropolymer photoresists. The incorporation of HFIP, with its strong electrophilic property and the electron-withdrawing effect of the fluorine atoms, significantly increases the acidity of the photoresist after exposure, enabling imaging without conventional photoacid generators (PAGs). The HFIP-containing photoresist has been evaluated by electron beam lithography to achieve a trench of ~40 nm at an extremely low dose of 3 μC/cm^2^, which shows a sensitivity enhancement of ~10 times compared to the commercial system involving PAGs, revealing its high sensitivity and high-resolution features. Our results demonstrate a new type of PAGs and a novel approach to higher-performance imaging beyond conventional photoresist performance tuning.

## 1. Introduction

Photolithography utilizes light to project a designed pattern onto a photosensitive photoresist, and the light sensitivity of photoresists is of crucial importance, as it influences the efficiency of the lithography and ultimately determines the yield of semiconductor manufacturing [1]. Extreme ultraviolet (EUV) lithography [2,3] and electron beam lithography (EBL) [4,5,6] possess state-of-the-art nanofabrication capabilities; however, stringent sensitivity requirements need to be met while maintaining the ability to print ever-shrinking feature sizes [7,8,9]. For the time being, this remains a challenge.

Researchers have recently developed a variety of candidate photoresist materials, including chemically amplified photoresists (CARs) [10,11,12], metal-based materials [13,14], and cleavage polymer-based non-CARs [15,16,17]. Of these, polymer-based CARs are considered the latest qualifying EUV photoresist for realizing high-volume manufacturing [18]. CARs involve resin with acid-sensitive leaving groups and PAGs which generate acids, such as trifluoromethanesulfonic acid, during exposure [19,20,21]. The photoacid acts as a catalyst to catalyze the deprotection reaction; moreover, it has the effect of chemical amplification and significantly improves the UV lithography sensitivity [22,23]. However, when conventional CARs are applied to the EUV platform, the sensitivity is severely affected by the limited EUV absorption.

Various attempts at creating highly sensitive EUV photoresists using CARs have been made, yet most of these focused on the optimization of acid-sensitizing groups and acid-producing agents. However, with the addition of PAGs, a paradox arises, where more PAG will significantly increase the sensitivity, yet the accompanying inevitable acid diffusion will deteriorate the resolution. To address this issue, Ober et al. [24] reported PAG-tethered self-immolated polymer resists and proposed that the restricted diffusion of photo-generated acid to the reactive polymer backbone contributed to a 3–4-fold enhancement in sensitivity. Researchers have also developed a variety of alternative materials by introducing metals [25] and halogens [26,27]. Yamamoto et al. [28] improved the sensitivity by 43% by adding a metal sensitizer and suggested that the improvement in sensitivity was mainly dependent on the increase in the yield of photoacid and photoelectrons. Jiang et al. [29] demonstrated that sensitizers containing F and I provide a significant advantage in absorption and electron generation, but the chemical environment where the halogens are bonded has a major impact on the sensitivity. These studies have provided critical insights into the design of EUV photoresists to improve photosensitivity. However, radiation-induced chemistry is not well understood, and it is difficult to associate them directly with the solubility switches through only the absorption properties that are exhibited by metals and halogens.

Trials to enhance photosensitivity have been conducted using a main chain break [30] or side-group-bonding PAGs [31], the addition of a crosslinker [32] or photosensitizing groups [33], and other approaches. Small molecules (star-type materials or molecular glass) and metal photoresists are also studied, aiming for high sensitivity and high resolution [14,15]. Ober’s group and Giannelis’ group [34] reported the first HfO_2_-based metal nanoparticle photoresists and studied them for use in electron beam lithography to obtain 50 nm lines with sensitivity of about 103 µC cm^−2^. Yang’s team [35,36] developed a chemically amplified photoresist by modifying the number of leaving groups to adjust its sensitivity to 52 µC cm^−2^. Numerous studies [10,37,38] have reported electron beam exposure doses of 10 µC/cm^−2^ or more for photoresists, with commercially available photoresists requiring as high as several hundred to thousands of µC cm^−2^ exposure doses [39,40,41].

**Scheme 1:**
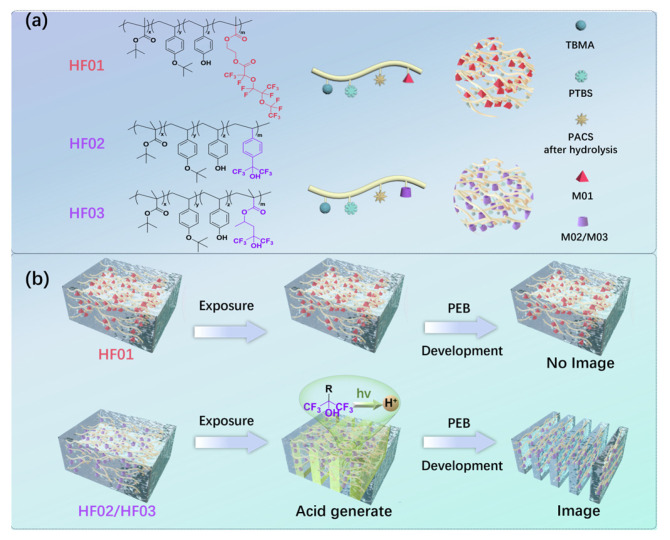
Strategy for fluoropolymer photoresist patterning. (**a**) Approach to synthesizing fluoropolymers (HF01~03): the copolymerization of tert-butyl methacrylate (TBMA), p-acetoxystyrene (PACS), p-tert-butoxystyrene (PTBS), fluorinated monomers 2-((2,3,3,3-tetrafluoro-2-(1,1,2,3,3,3-hexafluoro-2-(perfluoroethoxy)propoxy)propanoyl)oxy)ethyl methacrylate (M01), alpha, alpha-bis(trifluoromethyl)-4-vinylbenzyl alcohol (M02), and 1,1,1-trifluoro-2-trifluoromethyl-2-hydroxy-4-pentyl methacrylate (M03). (**b**) Comparison of lithographic imaging performance of photoresists using different fluoropolymers.

Here in our study, inspired by the high EUV absorption and strong electrophilic property that are provided by F, we have developed an innovative approach to highly sensitive nanopatterning with a series of fluoropolymer photoresists (Scheme 1). We were surprised to find that the HFIP in photoresists can serve as an acid-generating agent and achieve patterning, which could lead to a broad research field on PAGs and enrich the CAR family. This acid-producing mechanism was validated in both UV and EBL exposure approaches. The HFIP-containing photoresist shows superior patterning ability versus conventional PAG-involved systems with exposure doses of down to 3 μC/cm^2^, which is almost the minimum dose of the equipment. To the best of our knowledge, no research has been conducted to demonstrate HFIP serving as an acid-generating agent during exposure and significantly boosting the sensitivity. Our work will open a new way to higher-performance imaging beyond conventional formulation tuning.

## 2. Materials and Methods

The synthesis of copolymers was performed by the free radical polymerization of monomers in 1,4-dioxane as the solvent and azobisisobutyronitrile as the radical initiator at 80 °C for 6 h in a nitrogen atmosphere, followed by ammonolysis [42] of acetoxy groups to phenolic hydroxyl groups with ammonia water (Figure S1). The structures of the fluoropolymers (abbreviated as HF01~03) are shown in Scheme 1a. The Fourier transform infrared (FT-IR) spectra and the proton nuclear magnetic resonance (1H-NMR) spectra are drawn in Figures S2–S5 and Figures S6–S13, respectively. Fluorinated monomers mainly include those without (M01) and with (M02 and M03) HFIP structures. Gel permeation chromatography (GPC) analysis revealed that the Mw (average molecular weight) of the fluoropolymers ranged from 10,000 to 13,000 g/mol. Meanwhile, HF02 and HF03 exhibited superior polymerization efficiency and dispersion (Figure S14 and Table S1). The fluoropolymers showed good thermal stability (the 5 wt.% weight loss is at approximately 200 °C, as shown in Figures S16 and S17) and an outstanding alkali resistance and film retention rate (Figure S15).

## 3. Results

The preliminary EBL results showed that the HFIP-containing photoresists (HF02 and HF03) could be patterned, even without the addition of conventional PAGs, while the photoresists with fluoropolymer HF01 in the formulation had no sensitivity under similar conditions. As illustrated in Scheme 1b, we want to propose that the introduction of an HFIP structure will not only enhance the absorption and electron yield but also participate in chemical reactions which are critical to achieving imaging. To further investigate the imaging properties, we designed a variety of photoresists (abbreviated as S01~15) and evaluated them utilizing different exposure approaches, and the results are summarized in Table 1.

**Figure 1:**
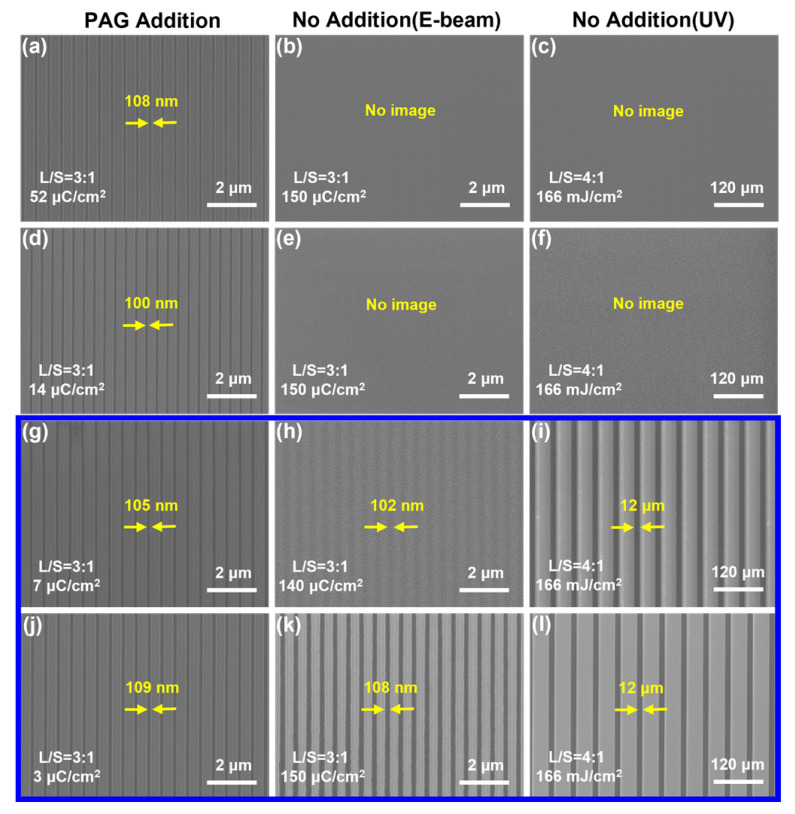
Comparison of UV and EBL performance of fluoropolymer-based photoresists with and without addition of conventional PAGs. (**a**,**d**,**g**,**j**) Photoresists S01~04 composed of polymers HF00, HF01, HF02, and HF03 and PAGs, respectively. (**b**,**c**) S05, (**e**,**f**) S06, (**h**,**i**) S07, and (**k**,**l**) S08 are photoresists composed of polymers HF00, HF01, HF02, HF03, respectively. Pitch and L/S of the designed layout are 400 nm and 3/1 (for EBL) and 60 μm and 4/1 (for UV), respectively.

The scanning electron microscopy (SEM) images of photoresists S01~04, exposed by EBL, showed clear line and space patterns, with a trench of approximately ~100 nm (Figure 1a,d,g,j). Compared to S01 using a non-fluorinated polymer, photoresists S02~04 using fluorinated polymers exhibited higher sensitivity, with the optimized dose reduced from 52 µC/cm^2^ to less than 14 µC/cm^2^, among which the doses for S03 and S04 were even lower at 7 µC/cm^2^ and 3 µC/cm^2^, respectively. We also evaluated the imaging properties of photoresists S05~08 without the addition of PAGs. The results showed that the photoresists using fluorine-free polymer (S05) and HF01 (S06) were unable to obtain line patterns (Figure 1b,e), while photoresists S07~08 with HFIP structures could achieve imaging (Figure 1h,k) under identical exposure conditions; especially, S08 (using HF03) demonstrated a better resolution (Figure 1k). However, the optimized dose was shifted to approximately 150 µC/cm^2^. In addition, this phenomenon was further verified during the UV exposure. After being exposed to a UV lamp (254 nm) for 166 mJ/cm^2^, the HFIP-containing photoresists (S07~08) yielded clear line patterns (Figure 1i,l), while photoresists S05~06 provided no image (Figure 1c,f). Utilizing HF00 and BJ3015 and HFIP-containing materials such as fluorinated polymers (HF02 and HF03) and monomers (M02 and M03), we also prepared several two-component photoresists (S09~15), which all gave discernible patterns (Figure S18). Since this type of photoresist follows the imaging mechanism of acid-induced deprotection, we propose that upon irradiation, the introduced HFIP structure can sever as an acid generator, significantly increasing the acidity of the exposed photoresist film area and subsequently catalyzing the deprotection to generate a positive-tone image.

**Figure 2:**
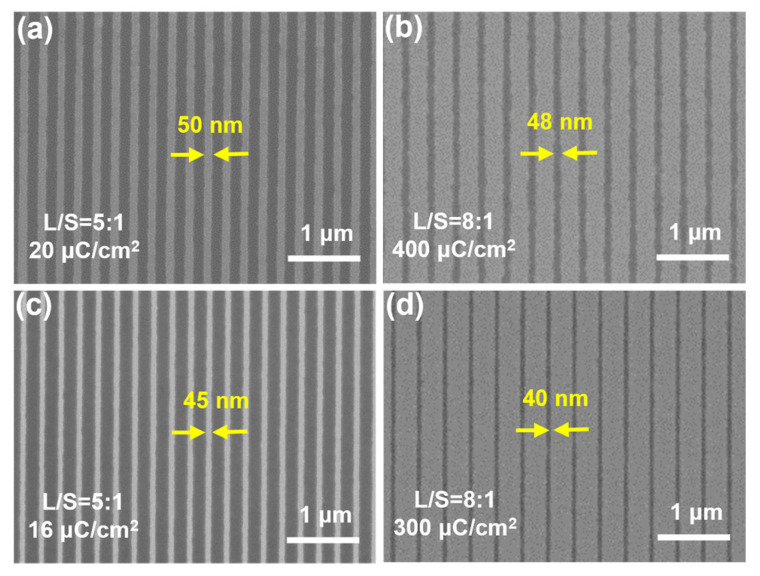
SEM images of photoresist patterns with a small feature size of sub-50 nm. (**a**) S03 and (**c**) S04 are photoresists using HF02 and PAGs and HF03 and PAGs, respectively. (**b**) S07 and (**d**) S08 are photoresists using HF02 and HF03, respectively.

We further evaluated the ability of such fluoropolymer photoresists to achieve smaller feature sizes. The results showed that line and space patterns with feature sizes down to 40 nm could be obtained by optimization (Figure 2d). Compared with the single-component fluoropolymer photoresist (Figure 2b,d), the two-component photoresists composed of fluoropolymer and PAGs (Figure 2a,c) showed significant advantages in terms of sensitivity and roughness, which may result in a higher contrast in development.

**Figure 3:**
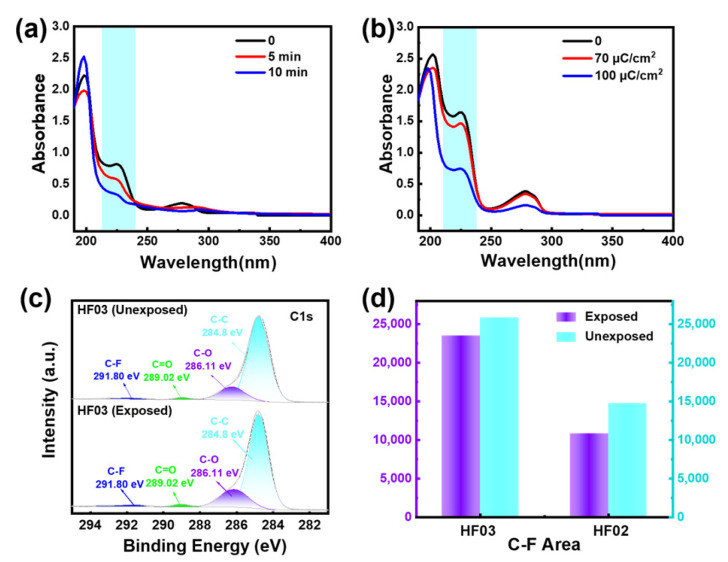
UV and XPS investigations. UV absorbance spectra of films composed of HF03 and a pH indicator bromocresol green before and after exposure to (**a**) UV light and (**b**) electron beam (30 kV). (**c**) C 1s spectra of HF03 film before and after exposure to electron beam (30 kV, 100 µC/cm^2^). (**d**) Calculated integral area of C-F from (**c**).

**Scheme 2:**
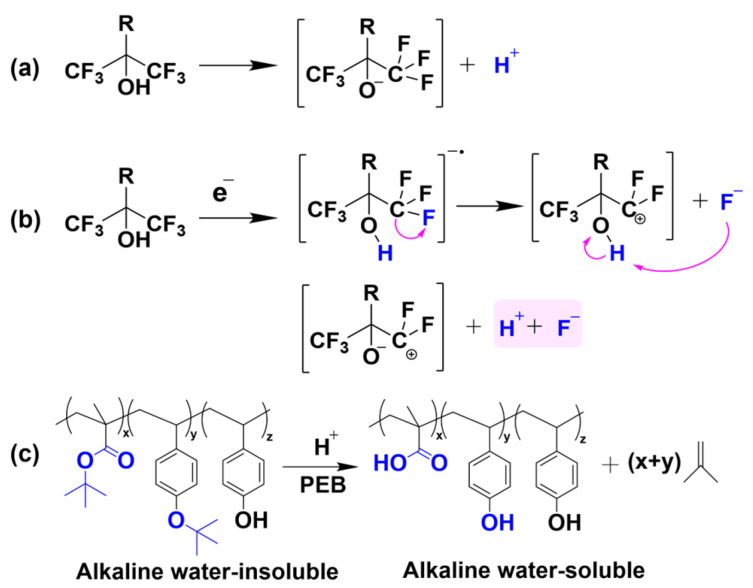
Proposed imaging mechanism of HFIP-containing fluoropolymer photoresists. (**a**) Dissociation of hydrogen ions. (**b**) Electrolytic dissociation and C-F bond cleavage, showing the formation of HF. (**c**) Acid-induced deprotection reaction.

To further investigate their acid-producing properties, we added a pH indicator bromocresol green into the fluoropolymer film and measured the change in UV absorption. An increase in acidity will cause the indicator’s absorption at 220 nm to be reduced due to conjugate addition [43,44] (Figures S19 and S20). As decided in Figure 3a,b, after UV or electron beam irradiation, there was a significant decrease in the absorbance at around 220 nm, resulting in a change that was greater than 50%, which is attributed to the proton gain. It was observed that the exposed polymer can release strong acids, which are critical for the deprotection of photoresist resins. Combined with the above lithography results, only the HFIP-containing fluoropolymer photoresists could be imaged, suggesting that HFIP is the source of acid generation after exposure. The X-ray photoelectron spectroscopy (XPS) results indicated a 35% decrease in C-F (291.8 ± 0.25 eV) [45] and a 14% decrease in C-O (286.1 ± 0.21 eV) for the exposed film compared to the unexposed film (Figures S21–S26), suggesting the cleavage of the C-F bonds and the deprotection of tert-butoxy groups. Therefore, we hypothesize that the C-F bonds in polymers are involved in chemical reactions in the presence of secondary electrons, and the chemical environment that is generated by C-F cleavage and the dissociation of hydrogen ions in the HFIP groups contribute together to the solubility transition caused by the acid-induced deprotection in the exposed region (Scheme 2c).

The well-known dissociative electron attachment (DEA) reaction is often reported for halogen-containing molecules [46,47]. The dissociative electron attachment reaction involves not only simple bond cleavage, but also multiple bond cleavages, rearrangement of precursor ions, and the formation of new molecules, in which the electron attachment proceeds through the formation of transient anions [48]. As illustrated in Scheme 2a,b, attributed to the electron-withdrawing -CF3 groups located close to the hydroxyl group, the HFIP group could effectively capture thermal electrons, easily excitable by electrons [49,50,51,52], and dissociates to generate H+ and anion fragments, leading to acid formation and an enhancement in acidity. Meanwhile, electron ionization events lead to the breaking of the C-F bond and the production of F−, whose nucleophilic attack will also accelerate the dissociation of H+. The presence of F− and H+ produces hydrofluoric acid (HF), which is much stronger than trifluoromethanesulfonic acid (a common photo-generated acid), leading to highly efficient acidogenic deprotection of tert-butoxy (Scheme 2c), ultimately enabling higher sensitivity.

## 4. Conclusions

In summary, we have designed and synthesized a novel type of HFIP-containing fluoropolymer photoresists and revealed the combination of the high absorption property provided by F, the strong electron-withdrawing effect of -CF_3_, and the cleavage of the C-F bond in electron ionization events, which will all contribute to the significant enhancement in sensitivity. Upon irradiation, the HFIP structure can dissociate to generate a strong acid and amplify the acidolytic deprotection reaction. Thanks to this, monomers or copolymers containing the HFIP structure can act as PAGs in CA photoresists. These special PAGs could be used together with traditional PAGs and open new opportunities to tune the performance of photoresists. This work may not only structurally enrich the family of PAGs and photoresist resins but also provide insights into the rational design of the next-generation photoresists. Further studies on the understanding and application of such HFIP-containing systems will be carried out.

## Figures and Tables

**Table 1:** Composition of photoresist samples and corresponding imaging results.

Sample No.	Polymer	PAG ^b^	Additive	Exposure Approach ^a^	
				UV	Electron Beam
S01	HF00	3 wt.%	-	√	√
S02	HF01	3 wt.%	-	√	√
S03	HF02	0.5 wt.%	-	√	√
S04	HF03	0.5 wt.%	-	√	√
S05	HF00	-	-	×	×
S06	HF01	-	-	×	×
S07	HF02	-	-	√	√
S08	HF03	-	-	√	√
S09	BJ3015	-	-	×	×
S10	BJ3015	-	HF02 ^c^	√	√
S11	BJ3015	-	HF03 ^c^	√	√
S12	BJ3015	-	M02 ^d^	√	√
S13	BJ3015	-	M03 ^d^	√	√
S14	HF00	-	M02 ^e^	△	-
S15	HF00	-	M03 ^e^	△	-

^a^ ×: Imaging cannot be achieved within UV exposure time of 10 min or electron beam dose of 400 µC/cm^2^. √: Imaging can be achieved under identical conditions. △: Weak sensitive imageability. ^b^ wt.% to polymer. ^c^ 5 wt.% to BJ3015. ^d^ 5 wt.% to BJ3015. ^e^ 2 wt.% to HF00.

## Data Availability

The data presented in this study are available on request from the corresponding author.

## Supplementary Materials

The following supporting information can be downloaded at https://www.mdpi.com/article/10.3390/polym16060825/s1. Figure S1: Material synthetic route of HF00, HF01, HF02, and HF03; Figure S2: FT-IR spectrum of the prepared copolymers HF00; Figure S3: FT-IR spectrum of the prepared copolymers of HF01; Figure S4: FT-IR spectrum of the prepared copolymers of HF02; Figure S5: FT-IR spectrum of the prepared copolymers of HF03; Figure S6: ^1^H NMR spectrum of the prepared copolymers of HF00 (400 MHz, DMSO-*d*_6_); Figure S7: ^1^H NMR spectrum of the prepared copolymers of HF01(400 MHz, DMSO-*d*_6_); Figure S8: ^1^H NMR spectrum of the prepared copolymers of HF02(400 MHz, DMSO-*d*_6_); Figure S9: ^1^H NMR spectrum of the prepared copolymers of HF03(400 MHz, DMSO-*d*_6_); Figure S10: ^13^C NMR spectrum of the prepared copolymers of HF00; Figure S11: ^13^C NMR spectrum of the prepared copolymers of HF01; Figure S12: ^13^C NMR spectrum of the prepared copolymers of HF02; Figure S13: ^13^C NMR spectrum of the prepared copolymers of HF03; Figure S14: GPC spectra of HF00, HF01, HF02, and HF03; Figure S15: Resist film loss of HF00, HF01, HF02, and HF03 after developing in 2.38 wt.% TMAH for different times; Figure S16: The TGA curves of copolymers HF00; Figure S17: The TGA curves of copolymers HF03; Figure S18: Comparison of E-beam performance of the fluoropolymer-based photoresists with the addition of HFIP monomer; Figure S19: UV absorbance spectra of HF00, HF01, HF02, and HF03; Figure S20: UV absorbance spectra of pH indicator bromocresol green with the increase of acid content; Figure S21: XPS C 1s spectra of S07 film; Figure S22: XPS C 1s spectra of S07 film after exposure to electron beam; Figure S23: XPS F 1s spectra of S08 film; Figure S24: XPS F 1s spectra of S08 film after exposure to electron beam; Figure S25: XPS F 1s spectra of S07 film; Figure S26: XPS F 1s spectra of S07 film after exposure to electron beam; Figure S27: LER and LWR of samples S01, S02, S03, and S08 at the exposure of an electron beam with a designed space width of 100 nm(L/S = 3:1) and 40nm (L/S = 8:1); Table S1: Characterization of molecular weight and polydispersity index (PDI); Table S2: The XPS peak position, area, and full width at half maximum (FWHM) of C 1s, O 1s, and F 1s of the unexposed and exposed photoresist film of S08; Table S3: The XPS peak position, area, and full width at half maximum (FWHM) of C 1s, O 1s, and F 1s of the unexposed and exposed photoresist film of S07; Table S4: The atomic % of C, O, and F of the unexposed and exposed photoresist film of S08 and S07, respectively; Table S5: LER and LWR values of samples S01, S02, S03, and S08. References [53,54,55,56] are cited in the Supplementary Materials.
