# Supplementary material for: Exceptional Lithography Sensitivity Boosted by Hexafluoroisopropanols in Photoresists
Source: Polymers (Basel). 2024 Mar 15;16(6):825. doi: 10.3390/polym16060825 (PMC10974190; doi:10.3390/polym16060825)
Supplement: Supplementary file 1 [file polymers-16-00825-s001.zip › polymers-2912630-supplementary.pdf]

Supporting Information

Exceptional Lithography Sensitivity Boosted by Hexafluoroisopropanols in Photoresists

Junjun Liu, Dong Wang, Yitan Li, Haihua Wang, Huan Chen, Qianqian Wang*, and Wenbing Kang*

National Engineering Research Center for Colloidal Materials, School of Chemistry and Chemical Engineering, Shandong University, Jinan 250100, Shandong, China

Corresponding author: wangqq2021@sdu.edu.cn; wbkang@sdu.edu.cn

Contents

Materials
Synthetic Procedures
Materials measurements
Lithography evaluation
Supporting Figures S1-S26
Supporting Tables S1-S4
References

Materials

The experimental raw materials, including p-acetoxystyrene (PACS), p-tert-butoxystyrene (PTBS), 1,1,1-Trifluoro-2-trifluoromethyl-2-hydroxy-4-pentyl methacrylate (M03), alpha, alpha -bis (trifluor omethyl)-4-vinylbenzyl alcohol (M02), 2-((2,3,3,3-tetrafluoro-2-(1,1,2,3,3,3-hexafluoro-2-(perfluoroe thoxy)propoxy)propanoyl)oxy)ethyl methacrylate (M01), and BJ3015 (resin containing vinyl ether protection) were obtained from Beijing Technology (Shandong) Co., Ltd. Additionally, tert-butyl methacrylate (TBMA, AR, 99%), propylene glycol monomethyl ether acetate (PGMEA, AR, 9 9%, containing 50 ppm BHT stabilizer), 1,4-dioxane (DO, AR, 99%), ammonia water (AR, 2 5%), and glacial acetic acid (AcOH, AR, 99%) were purchased from Shanghai Macklin Bioche mical Technology Co., Ltd (Shanghai, China). The azobisisobutyronitrile (AIBN, AR, 99%) was procured from Shanghai Dipper Biotechnology Co., Ltd (Shanghai, China). The water used in th e experiment was ultrapure.

Synthetic Procedures

Synthesis of HF00

The solvent of DO (66.51 g) was mixed in a round-bottom flask with AIBN (0.50 g) and heated to 80 °C under a nitrogen atmosphere with magnetic stirring. A mixture of PACS (11.35 g), TBMA (2.84 g), and PTBS (1.81 g) was added drop-wisely, and the reaction was stirred at 80 °C for six hours and cooled to room temperature. Then 27.2 g of ammonia water was added to the reaction mixture, then heated to 80 °C, and stirred for five hours. It was cooled to room temperature, and then 152.38 g of AcOH was added and stirred for two hours. The reaction mixture was precipitated into water at a 1:10 reaction mixture: water ratio. Then, the powder resin was filtered, washed with pure water, and dried in a vacuum oven to give HF00 resin in 90 % yield.

Synthesis of HF01

The solvent of DO (66.51 g) was mixed in a round-bottom flask with AIBN (0.50 g) and heated to 80 °C. Magnetic stirring was conducted at a rate of 30 rpm/min under a nitrogen atmosphere. Adding a mixture of PACS (11.35 g), TBMA (2.84 g), PTBS (1.81 g), and M01 (1.22 g), the reaction was heated for six hours at 80 ° C. The reaction is heated to 80 °C for five hours after adding 27.2 g of ammonia water and

stirring. It was reduced to room temperature following the hydrolysis, and then 152.38 g of AcOH was added and mixed for two hours. The reaction mixture was precipitated into water at a 1:10 reaction mixture: water ratio. Then, the powder resin HF01 was obtained in 81 % yield by filtration, washing, and drying in a vacuum oven.

Synthesis of HF02

The solvent of DO (66.51 g) was mixed in a round-bottom flask with AIBN (0.50 g) and heated to 80 °C. Magnetic stirring was conducted at a rate of 30 rpm/min under a nitrogen atmosphere. Adding a mixture of PACS (11.35 g), TBMA (2.84 g), PTBS (1.81 g), and M02 (0.53 g), the reaction was heated for six hours at 80 ° C. The reaction is heated to 80 °C for five hours after adding 27.2 g of ammonia water and stirring. It was reduced to room temperature following the hydrolysis, and then 152.38 g of AcOH was added and mixed for two hours. The reaction mixture was precipitated into water at a 1:10 reaction mixture: water ratio. Then, the powder resin HF02 was obtained in 88 % yield by filtration, washing, and drying in a vacuum oven.

Synthesis of HF03

The solvent of DO (66.51 g) was mixed in a round-bottom flask with AIBN (0.50 g) and heated to 80 °C. Magnetic stirring was conducted at a rate of 30 rpm/min under a nitrogen atmosphere. Adding a mixture of PACS (11.35 g), TBMA (2.84 g), PTBS (1.81 g), and M03 (0.46 g), the reaction was heated for six hours at 80 ° C. The reaction is heated to 80 °C for five hours after adding 27.2 g of ammonia water and stirring. It was reduced to room temperature following the hydrolysis, and then 152.38 g of AcOH was added and mixed for two hours. The reaction mixture was precipitated into water at a 1:10 reaction mixture: water ratio. Then, the powder resin HF03 was obtained in 90 % yield by filtration, washing, and drying in a vacuum oven.

Materials measurements

A scanning electron microscope (SEM, S-4800, Hitachi of Japan) was utilized to examine the materials' morphology and structure. The materials were examined using a UV-Vis spectrophotometer (UV-Vis, Carry5000, Agilent, Palo Alto) and a Fourier transform infrared spectrometer (FT-IR, Tensor II, Bruker, Billerica, USA). UV-absorption measurements were used to characterize the photo-acid generation

properties of the resins to identify the changes in organic polymers. The Thin-Film Analyzer (F20 Thin-Film Analyzer, Filmetrics, San Diego, USA) is utilized to analyze the properties of a film by measuring the amount of light reflected from the film within a specific wavelength range and determining the thickness of the material. The atomic force microscope (AFM, BioScope Resolve) image measurement was performed in scanassit-air mode using the Bruker Dimension Icon instrument. X-ray photoelectron spectroscopy (XPS, X Per3 Powder) was conducted using a Thermo Scientific Escalab 250Xi spectrometer with Al Kα (hv = 1486.6 eV) as the radiation source. This method was employed to identify alterations in the polymer composition and to obtain data on elemental ratios and the chemical environments of all the elements. The average molar mass and molar mass distribution were determined by gel permeation chromatography (GPC, BI-MWA, Brookhaven Instruments Corporation, Holtsville, USA) on a Waters 410 GPC using polystyrene as the standard and tetrahydrofuran as the eluent. The spectra were measured using a Bruker AVANCE III 400 WB spectrometer. The ^{1}H-NMR spectra were recorded at a resonance frequency of 400 MHz with DMSO as the solvent, and the ^{13}C-NMR spectra were recorded at a resonance frequency of 100 MHz.

Lithography evaluation

The lithography performance of the fluoropolymers was evaluated according to the following process. 5 wt.% resist solution in PGMEA was filtered through a 0.2 μm filter, coated on the silicon substrate at 1200 rpm for 60 s, followed by a soft bake at 120 °C for 2 min to obtain a film with a thickness of 80 nm approximately. The exposure was performed by a UV lamp and an electron beam direct writing equipment (Raith GmbH, Pioneer Two, Germany) with an accelerating voltage of 30 kV and a beam current of 50 pA. The exposure was followed by post-baking at 130 °C for 2 min and developed in a 2.38 wt.% tetramethylammonium hydroxide (TMAH) aqueous solution for 60 s.

Supporting Figures

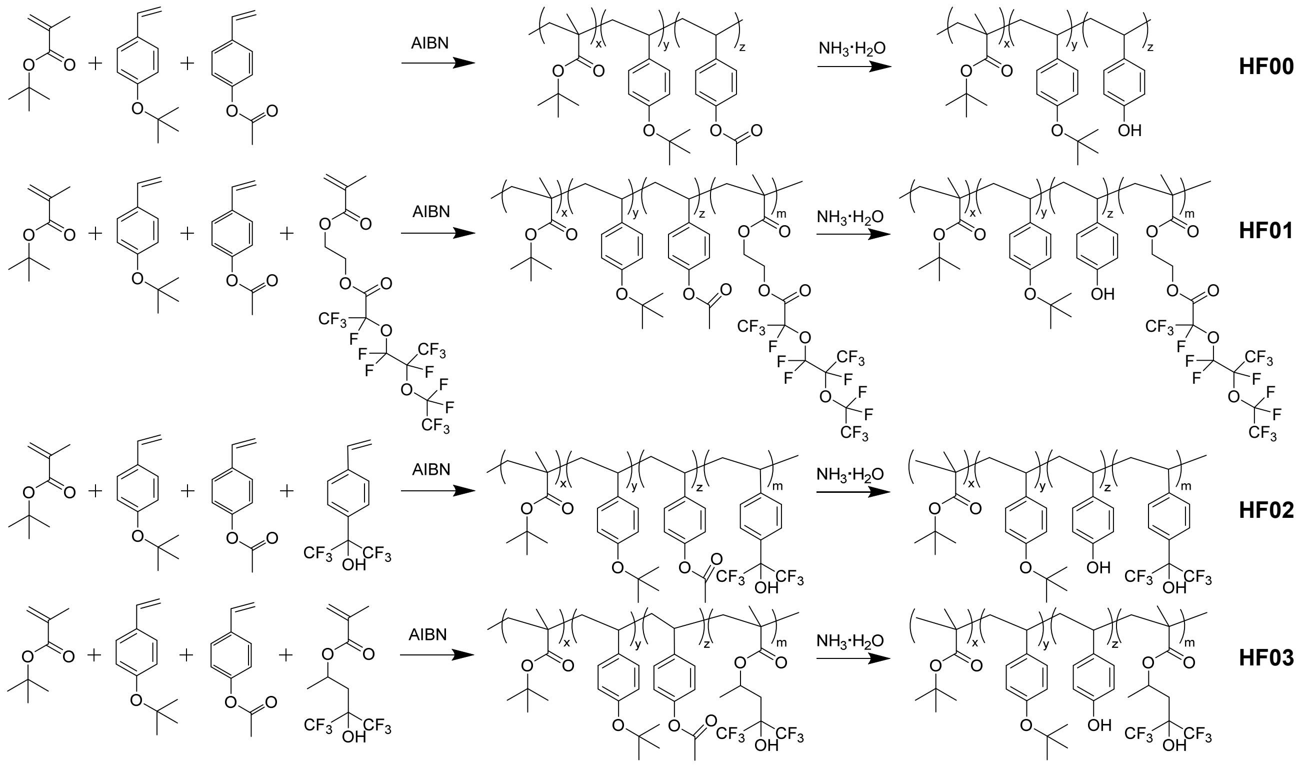

Figure S1. Material synthetic route of HF00, HF01, HF02, and HF03.

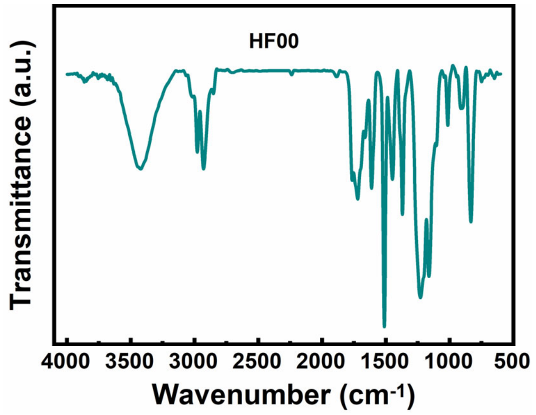

Figure S2. FT-IR spectrum of the prepared copolymers HF00.

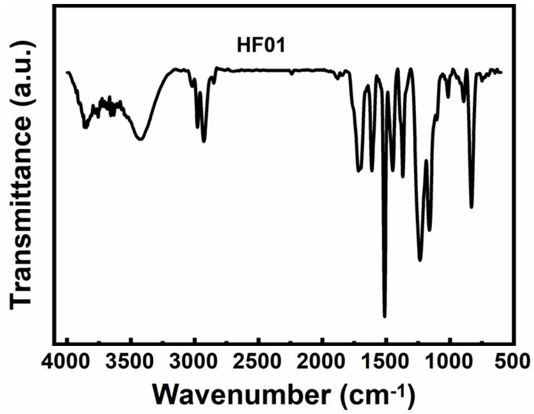

Figure S3. FT-IR spectrum of the prepared copolymers of HF01.

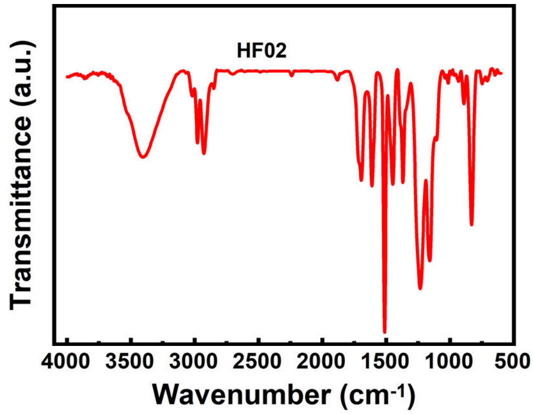

Figure S4. FT-IR spectrum of the prepared copolymers of HF02.

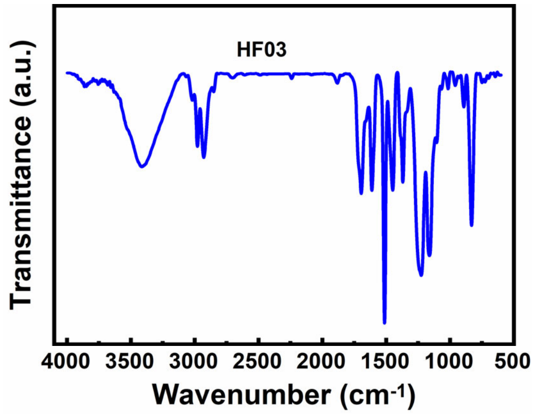

Figure S5. FT-IR spectrum of the prepared copolymers of HF03.

The infrared spectra of PHS resins and polymerized monomers with different fluorine structures are shown in **Figures S2-S5.** The peaks at 760 and 700 cm^{-1} are the out-of-plane bending vibration peaks of the benzene ring. The absorption peak near 3200-3000 cm^{-1} is caused by the stretching of unsaturated hydrogen in the benzene ring. The appearance of phenolic hydroxyl absorption at 3420 cm^{-1} corresponds

to the hydroxyl-OH telescoping vibration, which proves the hydrolytic deprotection of the precursor PACS. The peaks of the polymers HF00, HF01, HF02, and HF03 at 1717, 1716, 1694, and 1694 cm^{-1} correspond to the C=O stretching vibration, respectively[1]. Positional shifts correlate with intramolecular hydrogen bonding and isomerization of lipid structures. The peaks of HF00, HF01, HF02, and HF03 polymers at 2977 and 2928 cm^{-1} correspond to the stretching vibration couplings of the three C-H in methyl $-CH_3$, respectively, which demonstrates the successful polymerization of TBMA and PTBS. From 1110 cm^{-1} to 1200 cm^{-1} are the absorption peaks of the C-F[2], which overlaps with the C-O tensile vibration band.

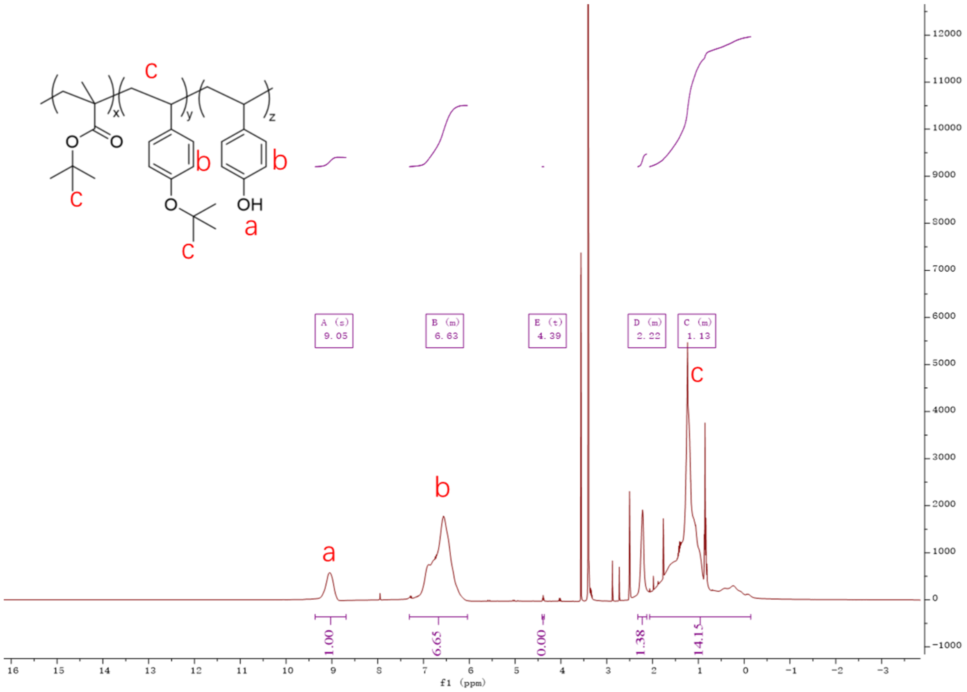

Figure S6. ^{1}H NMR spectrum of the prepared copolymers of HF00. (400 MHz, DMSO-d_6)

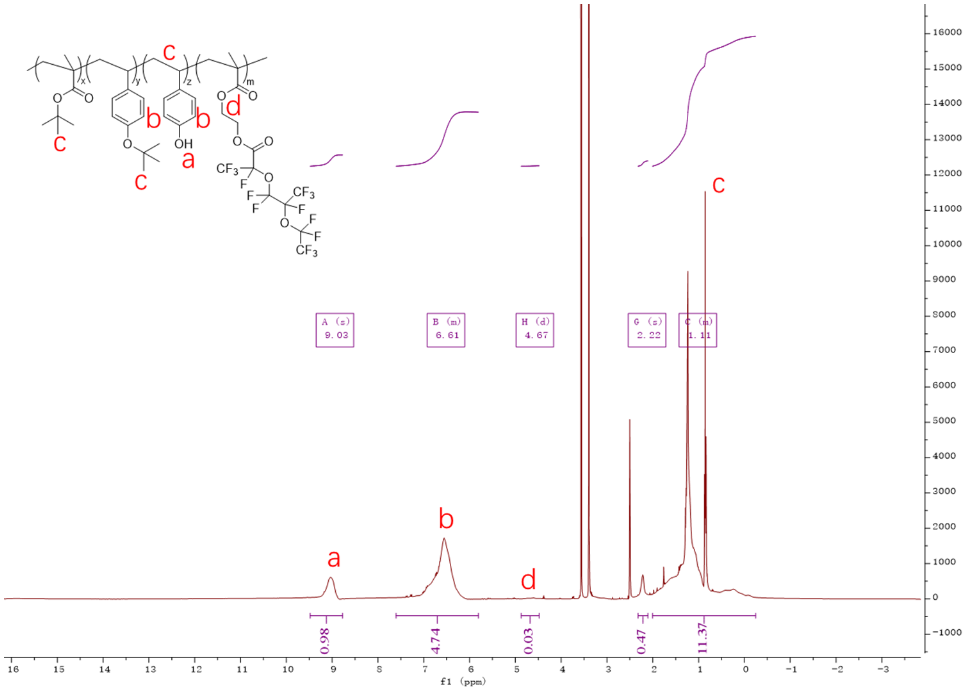

Figure S7. ^{1}H NMR spectrum of the prepared copolymers of HF01. (400 MHz, DMSO-d_6)

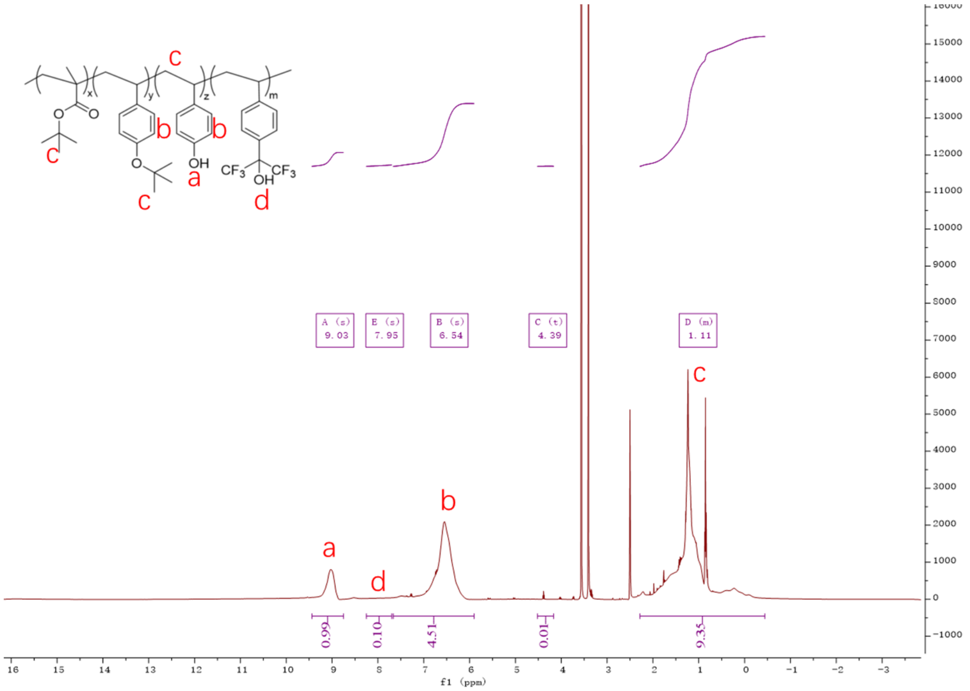

Figure S8. ^{1}H NMR spectrum of the prepared copolymers of HF02. (400 MHz, DMSO-d_6)

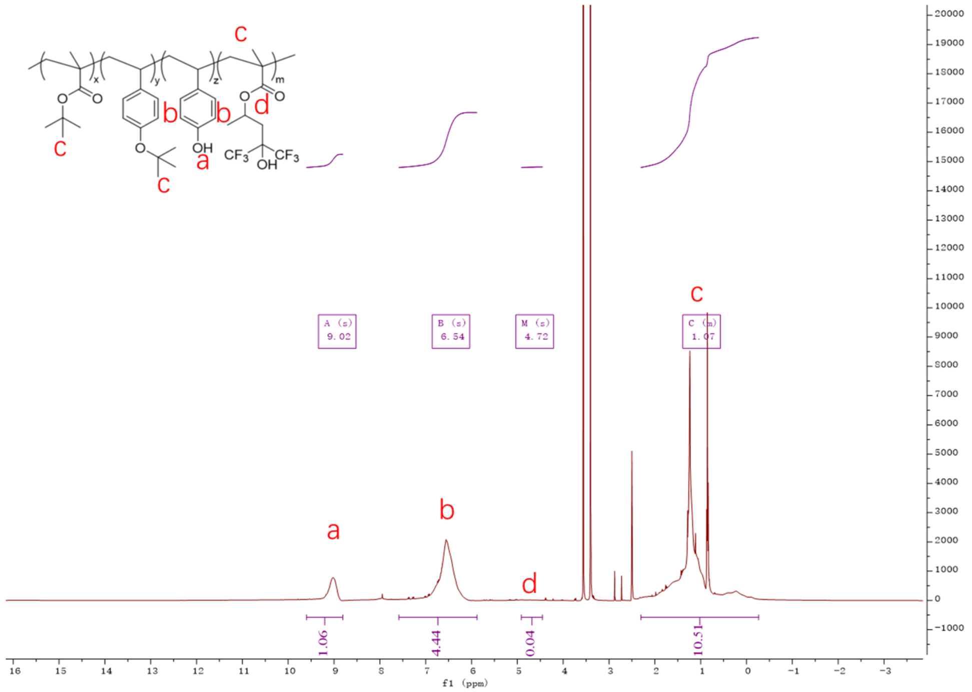

Figure S9. ^{1}H NMR spectrum of the prepared copolymers of HF03. (400 MHz, DMSO-d_6)

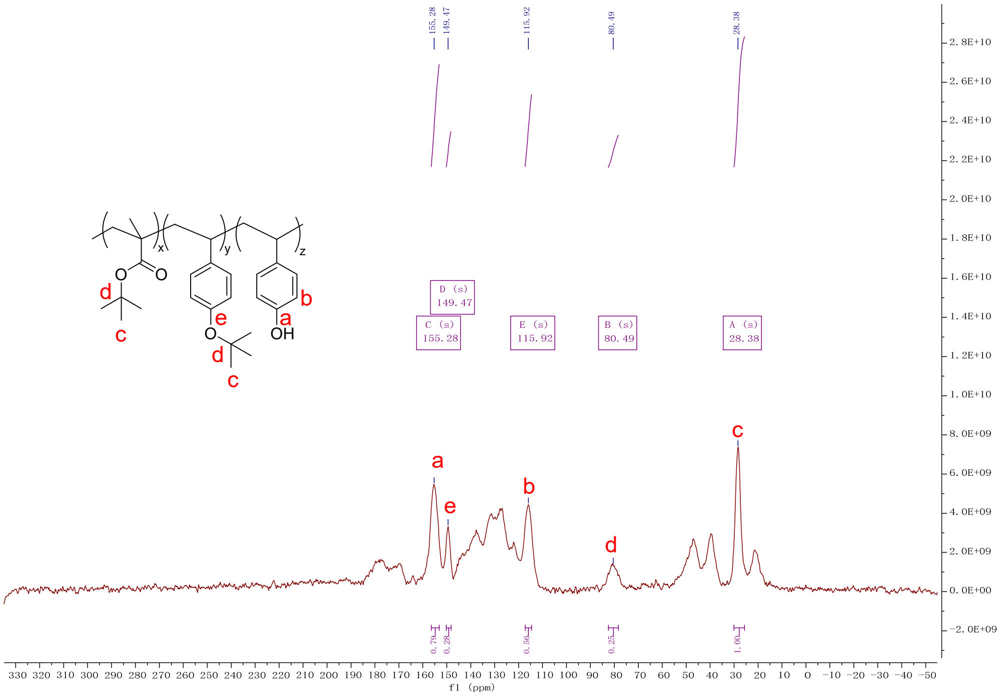

Figure S10. ^{13}C NMR spectrum of the prepared copolymers of HF00.

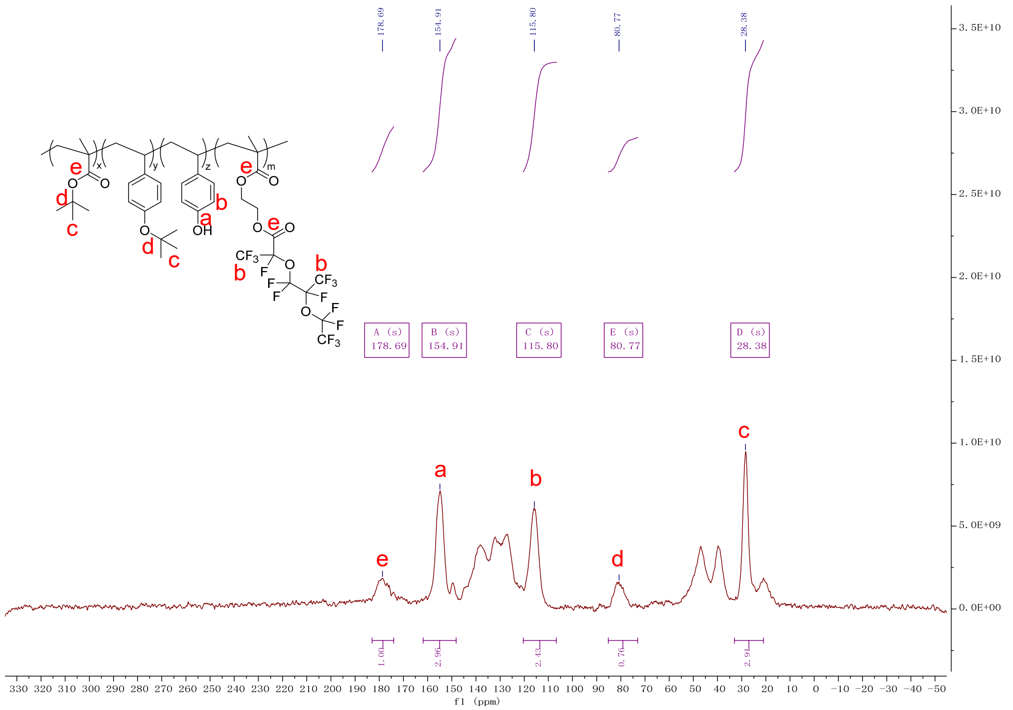

Figure S11. ^{13}C NMR spectrum of the prepared copolymers of HF01.

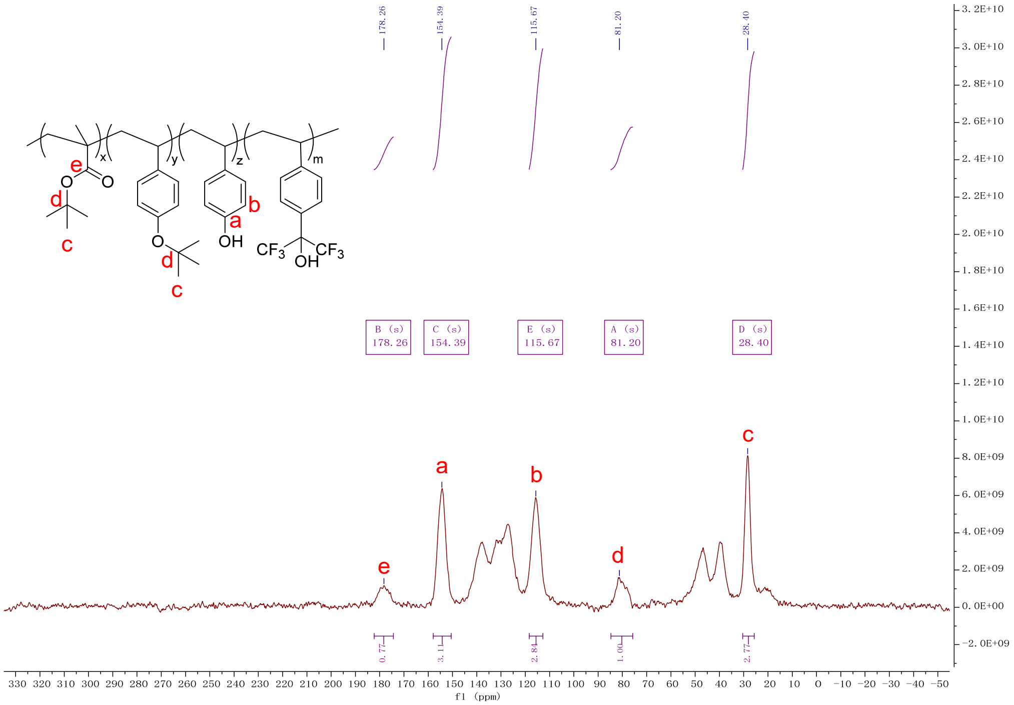

Figure S12. ^{13}C NMR spectrum of the prepared copolymers of HF02.

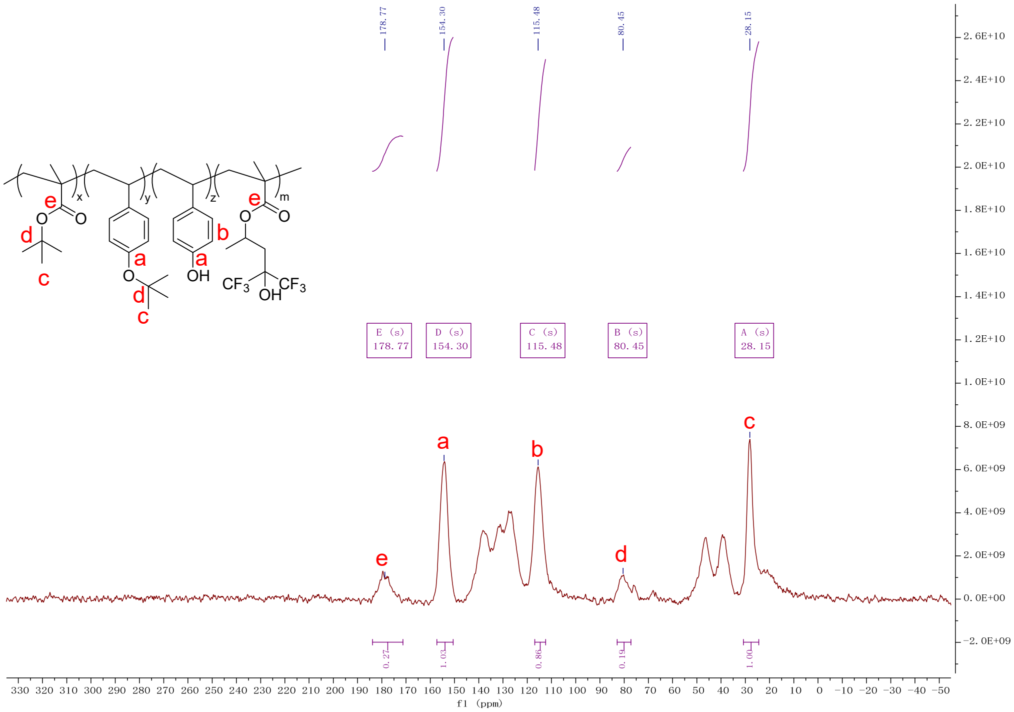

Figure S13. ^{13}C NMR spectrum of the prepared copolymers of HF03.

Figure S6-13 shows the spectra of NMR hydrogen and carbon spectra of four samples, HF00, HF01, HF02, and HF03, after ammonolysis, which can confirm the chemical structure of the polymers. From the NMR hydrogen spectrum, it was found that the signal peaks at 6~7.3 ppm originated from the aromatic protons. The signal peak at 9.0 ppm is significant and confirms the appearance of hydroxyl groups after ammonolysis[3-5]. There was no peak representing carboxylic acid hydrogens (generally >10.0 ppm), indicating the tert-butyl group on TBMA was not hydrolyzed under this hydrolysis condition. From the NMR carbon spectrum analysis, it was found that the carbonyl resonance corresponding to the carbonyl group at 167.4 ppm was removed due to the hydrolysis of ammonia, and the tert-butyl carbon corresponding to the carbonyl group at 80.0 ppm had an obvious signal peak, indicating the successful polymerization of the monomer, and the above NMR results corresponded to the IR results. Based on the peak areas attributed to the NMR hydrogen and carbon spectra, the molar percentage of the phenolic hydroxyl group after hydrolysis corresponding to the four samples, HF00, HF01, HF02, and HF03, was calculated to be 64.20%, 61.42%, 63.72%, and 67.91%, respectively. The hydrolysis efficiencies were

91.71%, 87.74%, 91.03%, and 97.01%, respectively, and the highest hydrolysis efficiency was found in HF03 among the four samples, which corresponded to the actual yield data of the samples.

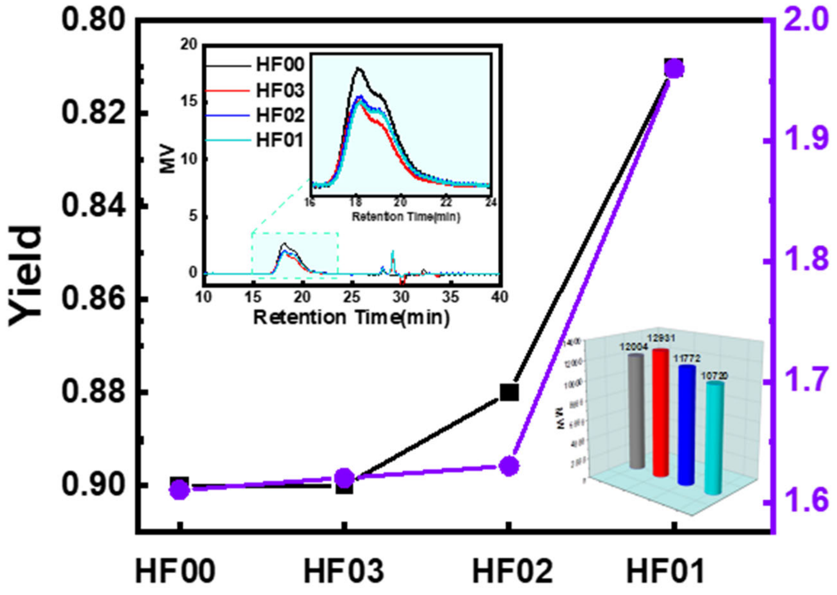

Figure S14. GPC spectra of HF00, HF01, HF02, and HF03.

In the GPC spectra shown in **Figure S14**, the peaks of the three curves at 18 min were the main polymer peaks, and the peaks near 30 min were mainly elution solvents. Comparison revealed that the dispersion of HF02, and HF03 in the samples after the polymerization of the three hexafluoroisopropanol monomers were smaller, and both of them had high polymerization efficiency and high reaction yields (**Table S1**).

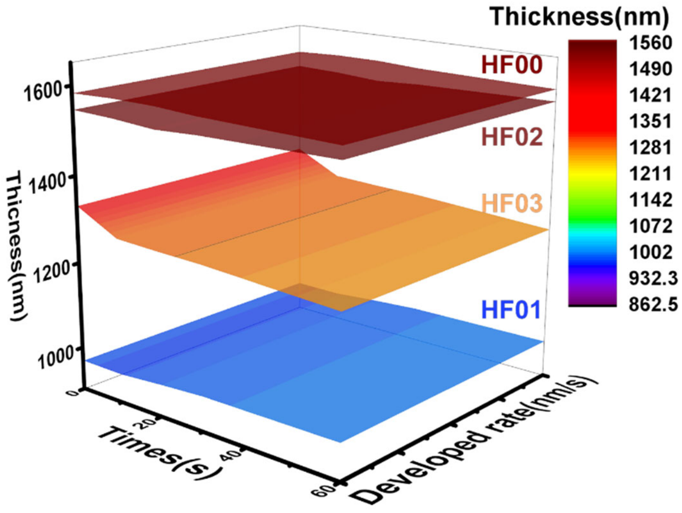

Figure S15. Resist film loss of HF00, HF01, HF02, and HF03 after developing in 2.38 wt.% TMAH for different times.

According to the film thickness change data of the samples under different development times, it can be seen that HF00, HF01, HF02, and HF03 have excellent alkali resistance and good film retention rates (**Figure S15**).

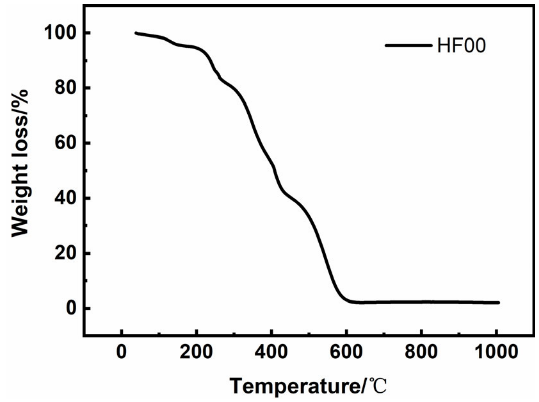

Figure S16. The TGA curves of copolymers HF00.

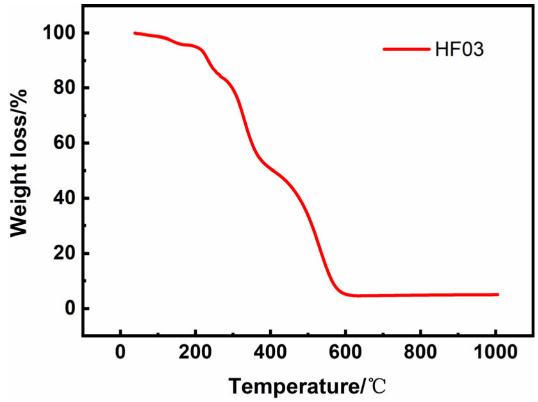

Figure S17. The TGA curves of copolymers HF03.

The thermal properties of the copolymers HF00 and HF03 are shown in **Figure S16-17**. All the copolymers generally were of high thermal decomposition temperature. The temperature of 5 wt.% loss of copolymers HF00 and HF03 were around 185 °C and 200 °C, respectively. The results indicated that all the samples in the nitrogen atmosphere are thermally stable below 200 °C.

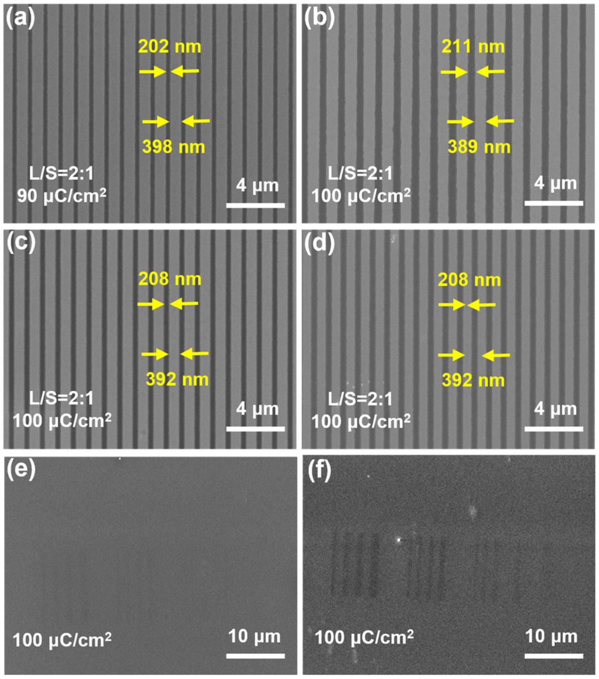

Figure 18. Comparison of E-beam performance of the fluoropolymer-based photoresists with the addition of HFIP monomer. (a) S10, (c) S11, (b) S12, and (d) S13 at the exposure of an electron beam with a designed space width of 200 nm (L/S = 2:1). (e) S14 and (f) S15 at the exposure of an electron beam with the dose of 100 μC/cm^2.

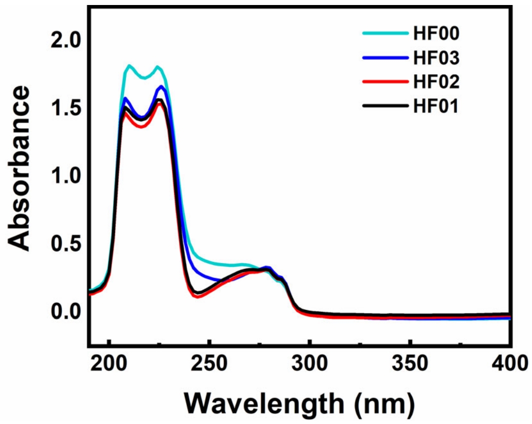

Figure S19. UV absorbance spectra of HF00, HF01, HF02, and HF03.

The chemical properties of the polymerized materials were further investigated by testing the changes in the UV-Vis absorption spectra of the four materials seen in **Figure S19**. The strongest absorption peaks at 208 nm, 225 nm, and 280 nm, respectively, were found from the UV spectra. The absorption bands are shown at 208 and 225 nm, which are caused by the electron π to π* jumps of the methacrylate in the material. In addition, the substitution of the auxochrome group -OH on the benzene ring of the material due to the n-π conjugation, and the presence of the two substituents of the disubstituted benzene in the para position and the electron-withdrawing group CF_3, all resulted in a larger redshift of the absorption band of E_2 and the enhancement of the absorbance[6, 7]. The strong absorption at 217 to 250 nm indicates the K absorption band with double bond and carbonyl group conjugation. The strong absorption at 250 to 300 nm indicates a B-band absorption, which is caused by the overlapping of the π→π* leaps in the conjugated system of three double bonds on the benzene ring and the vibrational phase of the benzene ring[8]. Above 300 nm it is almost transparent, so the four materials, HF00, HF01, HF02, and HF03, do not have conjugated systems in the molecules in the band above 300 nm.

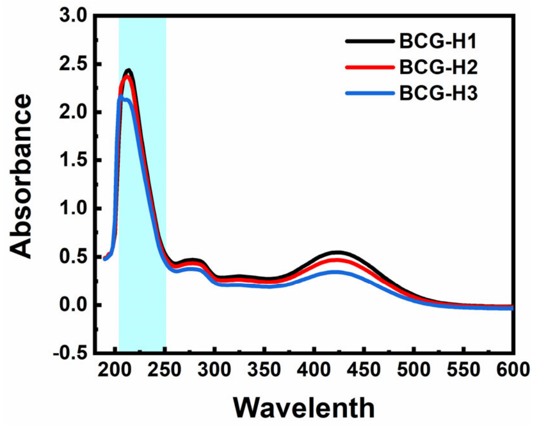

Figure S20. UV absorbance spectra of pH indicator bromocresol green with the increase of acid content.

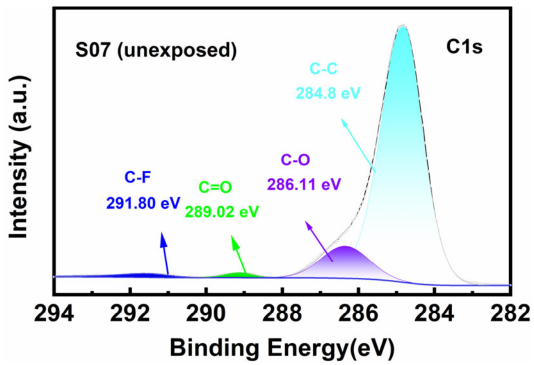

Figure S21. XPS C 1s spectra of S07 film.

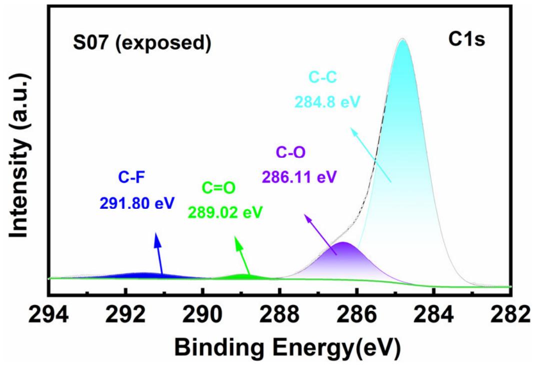

Figure S22. XPS C 1s spectra of S07 film after exposure to electron beam.

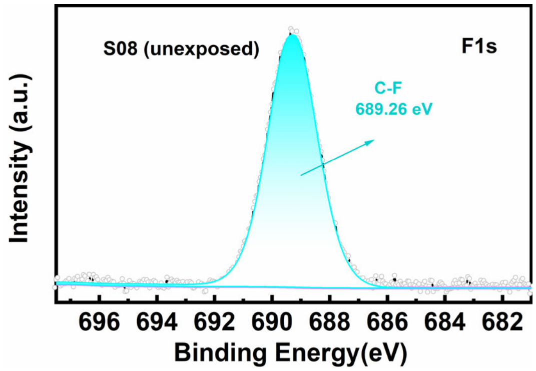

Figure S23. XPS F 1s spectra of S08 film.

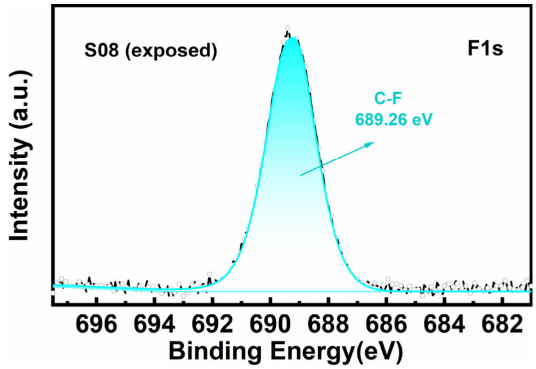

Figure S24. XPS F 1s spectra of S08 film after exposure to electron beam.

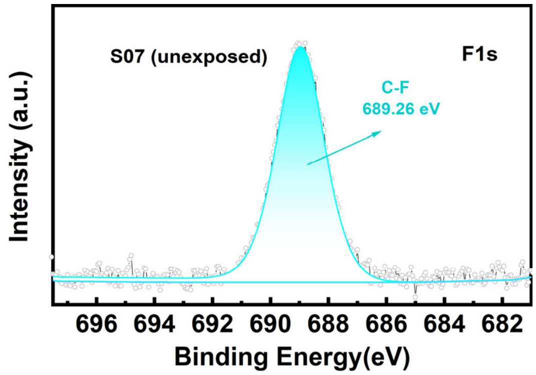

Figure S25. XPS F 1s spectra of S07 film.

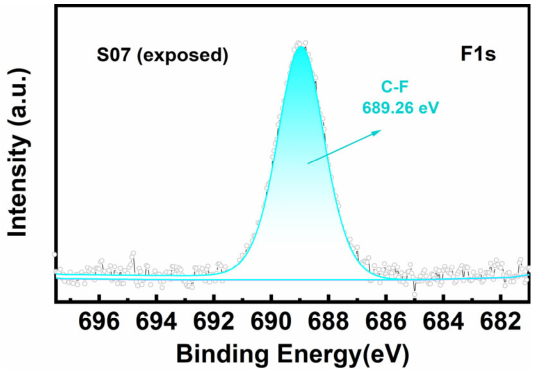

Figure S26. XPS F 1s spectra of S07 film after exposure to electron beam.

The C 1s spectra were fitted to obtain four components, and the higher oxidation state of the four components was analyzed, i.e., the C-F bond (292.8 eV) in which the C atoms are bonded to F. The peak at 688.5 eV was observed to be assigned to the fluoride ion in the high-resolution spectrum of F 1s, and the binding energy scale of each spectrum was calibrated using the above C 1s and C-F bonds as internal references. The C 1s spectra consisted of four fractions, as follows: sp^2 C (285.58 ± 0.25 eV), sp^3 C (286.41 ± 0.21 eV), COO (289.38 ± 0.22 eV), and C-F (292.81 ± 0.25 eV) [8].

Supporting Tables

Table S1. Characterization of molecular weight and polydispersity index (PDI).

Samples	M_W[a] (Daltons)	PDI	Yield
HF00	12004	1.61	90%
HF01	10720	1.96	81%
HF02	11772	1.63	88%
HF03	12931	1.62	90%

a. M_W: The average molecular weight of polymers.

Table S2. The XPS peak position, area, and full width at half maximum (FWHM) of C 1s, O 1s, and F 1s of the unexposed and exposed photoresist film of S08.

	Unexposed			Exposed		
Peak	Position	Area	FWHM	Position	Area	FWHM
C-C	284.80 eV	139397.03	1.35	284.80 eV	140522.02	1.35
C-O	286.11 eV	37043.73	1.35	286.11 eV	28257.87	1.35
C=O	289.02 eV	2623.36	1.35	289.02 eV	2345.19	1.35
C-F	291.80 eV	4673.53	1.35	291.80 eV	4095.84	1.35
F-C	689.26 eV	25836.71	1.90	689.26 eV	23507.43	1.90

Table S3. The XPS peak position, area, and full width at half maximum (FWHM) of C 1s, O 1s, and F 1s of the unexposed and exposed photoresist film of S07.

	Unexposed			Exposed		
Peak	Position	Area	FWHM	Position	Area	FWHM
C-C	284.80 eV	161153.69	1.35	284.80 eV	150195.63	1.35
C-O	286.11 eV	26865.42	1.35	286.11 eV	20869.91	1.35
C=O	289.02 eV	1861.12	1.35	289.02 eV	1781.02	1.35
C-F	291.80 eV	4794.08	1.35	291.80 eV	2822.15	1.35
F-C	689.26 eV	14704.89	1.90	689.26 eV	10803.82	1.90

Table S4. The atomic % of C, O, and F of the unexposed and exposed photoresist film of S08 and S07, respectively.

Element	Atomic%			
	S08		S07	
	Unexposed	Exposed	Unexposed	Exposed
C (total)	81.15	79.96	84.05	82.51
C-C	75.86	80.19	82.77	85.49
C-O	20.17	16.13	13.80	11.88
C=O	1.42	1.34	0.96	1.02
C-F	2.55	2.34	2.47	1.61
F (total)	3.69	3.45	1.92	1.89
O (total)	15.16	16.59	14.03	15.60

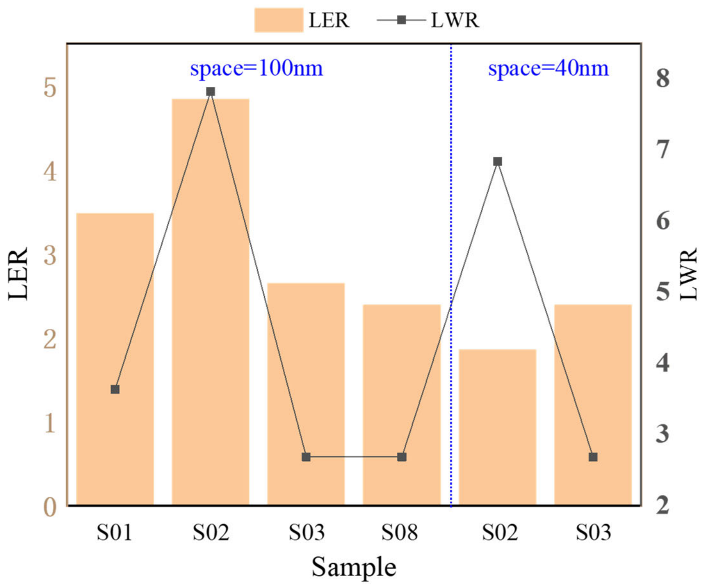

Figure S27. LER and LWR of sample S01, S02, S03, and S08 at the exposure of an electron beam with a designed space width of 100 nm(L/S = 3:1) and 40nm (L/S = 8:1).

Table S5. LER and LWR value of sample S01, S02, S03, and S08.

	width	L/S	LER	LWR
S01	100 nm	3:1	3.47	3.63
S02	100 nm	3:1	4.83	7.82
S03	100 nm	3:1	2.65	2.68
S08	100 nm	3:1	2.39	2.68
S02	40nm	8:1	1.85	6.84
S03	40nm	8:1	2.39	2.68

References:

(1) Li, H.; Liu, J.; Zheng, X.; Ji, C.; Mu, Q.; Liu, R.; Liu, X., Synthesis of chemically amplified photoresist polymer containing four (Meth) acrylate monomers via RAFT polymerization and its application for KrF lithography. *J. Polym. Res.* **2016,** *23* (5), 102-109.

(2) Thakur, N.; Vockenhuber, M.; Ekinci, Y.; Watts, B.; Giglia, A.; Mahne, N.; Nannarone, S.; Castellanos, S.; Brouwer, A. M., Fluorine-Rich Zinc Oxoclusters as Extreme Ultraviolet Photoresists: Chemical Reactions and Lithography Performance. *ACS Mater. Au* **2022,** *2* (3), 343-355.

(3) Yu, Y.-G.; Chae, C.-G.; Kim, M.-J.; Seo, H.-B.; Grubbs, R. H.; Lee, J.-S., Precise Synthesis of Bottlebrush Block Copolymers from ω-End-Norbornyl Polystyrene and Poly(4-tert-butoxystyrene) via Living Anionic Polymerization and Ring-Opening Metathesis Polymerization. *Macromolecules* **2018,** *51* (2), 447-455.

(4) Bae, Y. C.; Douki, K.; Yu, T. Y.; Dai, J. Y.; Schmaljohann, D.; Koerner, H.; Ober, C. K., Tailoring transparency of imageable fluoropolymers at 157 nm by incorporation of hexafluoroisopropyl alcohol to photoresist backbones. *Chem. Mater.* **2002,** *14* (3), 1306-1313.

(5) Chaffins, S.; Hinch, G.; DeKam, K.; Waterhous, V.; Smith, J.; Overbay, M.; Bilich, D.; Hovermale, C.; Jones, J., Epoxidized perfluoropolyethers: A route to hydrophobic, negative-tone photoresists. *J. Appl. Polym. Sci.* **2012,** *124* (6), 4636-4644.

(6) Wu, L.; Baljozovic, M.; Portale, G.; Kazazis, D.; Vockenhuber, M.; Jung, T.; Ekinci, Y.; Castellanos, S., Mechanistic insights in Zr- and Hf-based molecular hybrid EUV photoresists. *J. Micro/Nanolith. MEMS MOEMS* **2019,** *18* (1), 013504.

(7) Mattson, E. C.; Cabrera, Y.; Rupich, S. M.; Wang, Y.; Oyekan, K. A.; Mustard, T. J.; Halls, M. D.; Bechtel, H. A.; Martin, M. C.; Chabal, Y. J., Chemical Modification Mechanisms in Hybrid Hafnium Oxo-methacrylate Nanocluster Photoresists for Extreme Ultraviolet Patterning. *Chem. Mater.* **2018,** *30* (17), 6192-6206.

(8) Thakur, N.; Bliem, R.; Mochi, I.; Vockenhuber, M.; Ekinci, Y.; Castellanos, S., Mixed-ligand zinc-oxoclusters: efficient chemistry for high resolution nanolithography. *J. Mater. Chem. C* **2020,** *8* (41), 14499-14506.
